# Learning exceptions to the rule in human and model via hippocampal encoding

**DOI:** 10.1038/s41598-021-00864-9

**Published:** 2021-11-02

**Authors:** Emily M. Heffernan, Margaret L. Schlichting, Michael L. Mack

**Affiliations:** Department of Psychology, University of Toronto, Toronto, ON Canada

**Keywords:** Computational neuroscience, Learning and memory, Hippocampus, Human behaviour

## Abstract

Category learning helps us process the influx of information we experience daily. A common category structure is “rule-plus-exceptions,” in which most items follow a general rule, but exceptions violate this rule. People are worse at learning to categorize exceptions than rule-following items, but improved exception categorization has been positively associated with hippocampal function. In light of model-based predictions that the nature of existing memories of related experiences impacts memory formation, here we use behavioural and computational modelling data to explore how learning sequence impacts performance in rule-plus-exception categorization. Our behavioural results indicate that exception categorization accuracy improves when exceptions are introduced later in learning, after exposure to rule-followers. To explore whether hippocampal learning systems also benefit from this manipulation, we simulate our task using a computational model of hippocampus. The model successful replicates our behavioural findings related to exception learning, and representational similarity analysis of the model’s hidden layers suggests that model representations are impacted by trial sequence: delaying the introduction of an exception shifts its representation closer to its own category members. Our results provide novel computational evidence of how hippocampal learning systems can be targeted by learning sequence and bolster extant evidence of hippocampus’s role in category learning.

## Introduction

Category learning is a mechanism by which we make sense of the influx of information present in our daily lives. When we encounter a novel object or situation, we can compare it to previous experiences to make inferences about its qualities. Rapidly generalizing prior knowledge to new information is especially important when learning complex category structures. An example of such a problem is one in which categories are defined by a rule-plus-exceptions (RPE) structure where most category members adhere to a rule (e.g., birds fly), but a small subset of exceptions violates this rule (e.g., kiwis are flightless birds). A successful learner of RPE structure must detect general patterns across multiple experiences while also distinguishing and remembering irregularities. A wide body of evidence spanning several literatures indicates not only that RPE category rules are readily learned but also that rule-violating exceptions items are better remembered, potentially due to the formation of more detailed neural representations^1–6^.

Indeed, category learning is a complex process that recruits multiple brain regions^7^; however, recent work implicates hippocampus (HC) as a potential key player^5,8–11^. Notably, HC forms conjunctive representations of experiences that bind together multiple episodic features^12,13^. This rapid formation of conjunctive representations is especially important when learning complex category structures like in RPE learning. The formation of distinct conjunctive representations is consistent with traditional views of HC’s role in episodic memory formation^14^, but recent work has also implicated HC in rapid generalization across related experiences, as in statistical learning^15^ and complex associative memory^16,17^. HC’s ability to support these complementary processes may be attributed to two functional pathways that traverse specialized hippocampal subfields. Dentate gyrus (DG) and cornu ammonis 3 (CA3) fall along the trisynaptic pathway (TSP) and are associated with sparse representations appropriate for encoding distinct episodes; conversely, CA1, which is directly connected to entorhinal cortex (ERC) by the monosynaptic pathway (MSP), employs dense, overlapping representations ideal for extracting regularity^13,14,18,19^. The pattern separation from and generalization with prior experiences respectively enabled by TSP and MSP render this model of HC function well suited to support the divergent needs of RPE learning; however, the role of HC computations in such learning remains untested.

Intuitively, learning an exception to a rule requires first learning that rule. Indeed, it has been demonstrated that in an RPE task, learning sequences that group together rule-consistent stimuli apart from exceptions significantly improve overall learning outcomes compared to sequences that maximize or minimize between-trial similarity^20,21^. From the perspective of the HC model introduced above, the nature of prior experiences is key to how new information is encoded into organized memory structures, and this is particularly important when considering RPE learning. The prediction follows that CA1-supported generalization processes will capitalize on initial exposure to rule-consistent category members to form abstracted representations of rule-based category knowledge. When the learner later experiences exceptions, comparisons to these rule-based representations result in mismatch signaling^3,8,22^ that drives pattern-separation processes in DG and CA3 to distinctly encode exception information and facilitate learning^23^.

**Figure 1:**
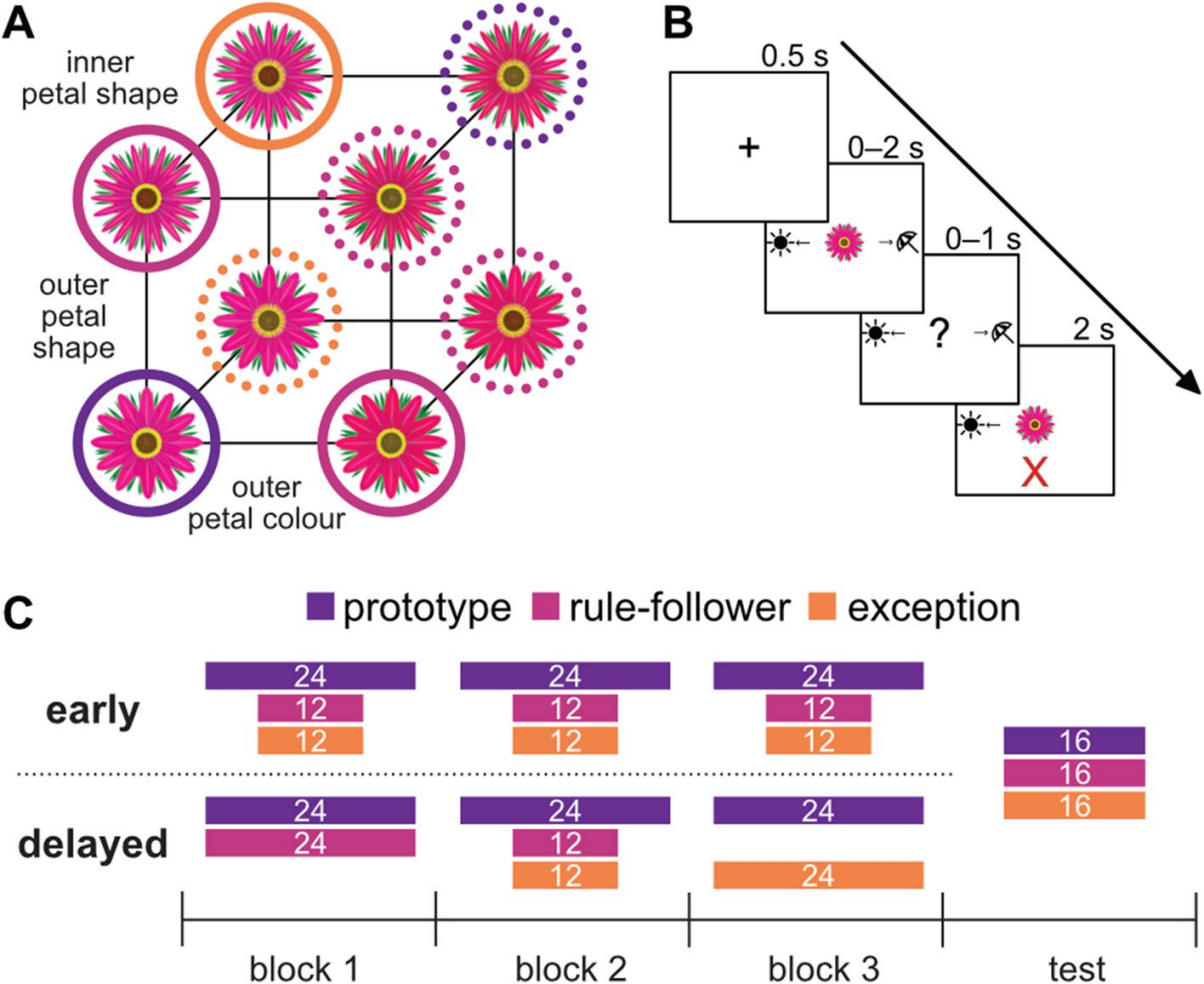
Experimental design. (**A**) Flowers were categorized using the Type III problem defined by^24^. Flowers varied across three binary-valued diagnostic dimensions and were defined as prototypes (circled in purple), rule-followers (circled in pink), and exceptions (circled in orange). Line type (solid or dashed) indicates category membership. (**B**) In learning trials, participants were exposed to stimuli and were asked whether the presented flower preferred sun or shade. They were required to make a category response within 2 s. After making a response, participants received feedback for 2 s before proceeding to the next trial. Testing trials followed a similar pattern, but no feedback was provided. The test block was followed by a recognition memory task (not shown). (**C**) The breakdown of stimulus types across conditions. The numbers of prototypes, rule-followers, and exceptions per block are denoted with purple, pink, and orange rectangles, respectively. Each block has 48 trials, and the width of the rectangles in (**C**) is proportional to the number of trials per type. In the “early” condition, participants saw exceptions in learning block one; in the “delayed” condition, exceptions were not introduced until the second learning block. The total number of stimuli and the structure of the test block were identical across conditions.

To explore the impact of learning sequence on categorization and to test how this manipulation may target key HC computations, here we devised a rule-plus-exception categorization task in which the introduction of exception items occurred either early in learning or later in learning, after extensive exposure to rule-following items. Participants learned to categorize flowers that varied across four binary-valued dimensions, three of which were diagnostic, and category structure followed the “Type III” categorization problem defined by Shephard et al.^24^, as shown in Fig. 1A. Each category had four members: one prototype, two rule-followers, and an exception. Prototypes from opposing categories were maximally dissimilar and varied across all three diagnostic dimensions, rule-followers varied from their category prototype across one dimension, and exceptions varied from their category prototype across two dimensions and were more similar to the prototype of the opposite category. In each category, two prototype variants were included, one for each value of the nondiagnostic dimension; the nondiagnostic dimension varied randomly for the remaining stimuli, resulting in a total of 10 stimuli. Participants completed three learning blocks with full feedback (Fig. 1B), followed by a test block in which they received no feedback. After the test block, participants completed a recognition memory task to test for differences in memory for exceptions compared to rule-following items. Participants were randomly separated into two conditions: in the baseline “early” condition, which matches the manner in which exceptions are typically introduced in RPE categorization tasks, participants were first exposed to exceptions in the first learning block, and in the “delayed” condition, exceptions were withheld until the second learning block. The test block was identical across conditions. A breakdown of stimuli in each block is presented in Fig. 1C.

We predicted that learners who were introduced to exceptions later in learning would develop a better initial representation of category structure, which in turn would improve their ability to detect and learn to categorize exceptions. In the context of hippocampal learning systems, in the delayed exceptions sequence, CA1 should capitalize on early experiences to capture the category structure. Mismatch signalling should then drive CA3 and DG to create unique representations of exception items when they are later introduced. To test whether HC is targeted by this sequence manipulation, we simulated this task with an extant neural network model of HC^15,25^. This model was designed to explore mechanisms behind HC’s role in episodic memory^25^, but recent work has demonstrated that this model also has the capacity for statistical learning^15^. If HC computations are sensitive to our manipulation, we predicted that the model would also be better able to categorize exception items when their introduction was delayed. As this model was not originally designed to model category learning, its successful replication of behavioural findings would provide compelling evidence of how hippocampal computations may support RPE learning. Existing models of category learning have successfully captured human behaviour in RPE tasks^5,26–28^, but to our knowledge, this work is the first to use a physiologically based model of HC to model such a task. We further conducted an exploratory representational similarity analysis of hidden layers of the neural network to explore how this manipulation impacted the model’s “neural” representations^15^. Because novelty facilitates the formation of specialized representations^3,8,22^, we expected to observe shifts in the model’s representations of exceptions introduced later in learning that were consistent with its performance. Such a finding would indicate that the distinctly encoded exceptions were better integrated into existing category knowledge. The behavioural experiment and a subset of the analyses were preregistered (https://osf.io/gner5); the model simulation approach was not. Deviations from our preregistered analysis plan are noted with justification throughout the results. For behavioural results that were preregistered but are not the focus of the paper, see the Supplement.

## Results

### Behavioural study

We first explored how delaying the introduction of exceptions would impact learning performance in young adults. We hypothesized that this delay would allow participants to form a strong understanding of the rules governing category structure compared to a baseline condition in which exceptions were introduced at the outset of learning. Specifically, when exceptions were introduced later in learning, enhanced mismatch signalling would allow participants to better identify and correctly categorize these exceptions. Participants were separated into “delayed exceptions” and “early exceptions” conditions, and we expected to see improved categorization of exception items in the delayed condition. We assessed this prediction in two separate analyses: one for the three learning blocks, and a second for the test block.

We analyzed learning performance with a binomial generalized linear mixed-effects (GLME) regression model to test the impact of stimulus type (exception, prototype, or rule-follower) and sequence (early or delayed) on accuracy, averaged across all three learning blocks. In all behavioural analyses, participant was included as a random effect.

Stimulus type had a significant effect on accuracy. In the early condition, categorization accuracy was significantly higher for rule-followers than exceptions (β_R:E_ = 0.829, *P* < 0.001, 95% CI [0.68, 0.98]), and accuracy was higher for prototypes than rule-followers (β_P:R_ = 0.541, *P* < 0.001, 95% CI [0.37, 0.71]—where subscripts E, P, and R denote exceptions, prototypes, and rule-followers, respectively). The same patterns held in the delayed condition (β_R:E_ = 0.362, *P* < 0.001, 95% CI [0.21, 0.51] and β_P:R_ = 1.211, *P* < 0.001, 95% CI [1.03, 1.39], respectively). Condition had a significant effect on categorization accuracy: accuracy was higher in the delayed compared to the early condition for both exceptions (β = 0.317, *P* = 0.001, 95% CI [0.13, 0.51]) and prototypes (β = 0.521, *P* < 0.001, 95% CI [0.30, 0.74]); no significant effect was found for rule-followers (β = − 0.150, *P* = 0.128, 95% CI [− 0.34, 0.04]). There were also interactions between type and condition. The difference in categorization accuracy between exceptions and prototypes did not significantly change across conditions (β = 0.203, *P* = 0.101, 95% CI [− 0.04, 0.45]), but the difference between exceptions and rule-followers was significantly lower in the delayed compared to the early condition (β = − 0.467, *P* < 0.001, 95% CI [− 0.68, − 0.25]), and the difference between rule-followers and prototypes was significantly higher in the delayed condition (β = 0.670, *P* < 0.001, 95% CI [0.42, 0.92]).

Having established overall differences in accuracy across all blocks in the learning task, we next asked whether change in performance over experience varied significantly as a function of item type and early/delayed introduction. We thus fit a second model to the learning data that contained repetition as a fixed effect. Repetition, which was defined as the number of appearances of a given stimulus type throughout the three learning blocks and is distinct from trial number, was included to explore how accuracy was affected at different timepoints throughout learning.

**Figure 2:**
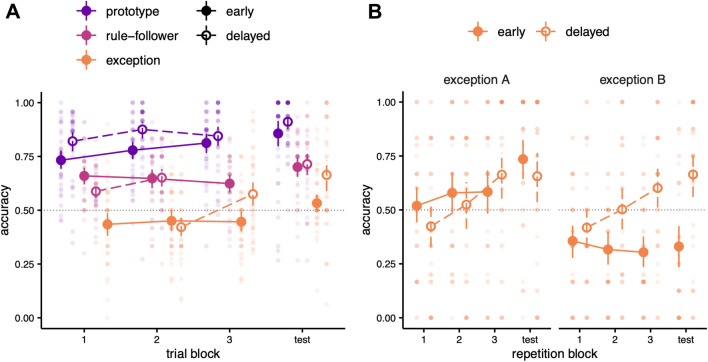
Categorization accuracy in the behavioural experiment. (**A**) Results from the three learning blocks (48 trials total per block) and the test block (48 trials total) for all stimuli. Large points indicate the median of bootstrapped mean accuracies for stimuli in each category across block and condition. Error bars represent bootstrapped 95% confidence intervals. Small dots show average accuracy for individual participants. Categorization accuracy improved more for exceptions in the delayed exceptions condition compared to the early condition. (**B**) Exception results, separated by category. Exception trials have been grouped into blocks of 12 (i.e., repetition block 1 encompasses the first 12 appearances of exceptions in the early or delayed condition).

In the delayed condition, there was a main effect of repetition: categorization accuracy for exceptions, prototypes, and rule-followers improved significantly with repetition (β_E_ = 0.038, *P* < 0.001, 95% CI [0.03, 0.05]; β_P_ = 0.037, *P* < 0.001, 95% CI [0.02, 0.05]; β_R_ = 0.019, *P* =  < 0.001, 95% CI [0.01, 0.03]. In the early condition, categorization accuracy for prototypes improved significantly with repetition (β_P_ = 0.017, *P* = 0.011, 95% CI [0.004, 0.03]); however, categorization accuracy for exceptions and rule-followers showed no significant improvement with repetition (β_E_ = 0.006, *P* = 0.275, 95% CI [− 0.01, 0.02]; β_R_ = − 0.010, *P* = 0.081, 95% CI [− 0.02, 0.001]). There was also an interaction between repetition and learning condition. Categorization accuracy improved more with increased repetition in the delayed condition than in the early condition for all stimulus types (β_E_ = 0.032, *P* < 0.001, 95% CI [0.02, 0.05]; β_P_ = 0.021, *P* = 0.036, 95% CI [0.001, 0.04]; β_R_ = 0.029, *P* < 0.001, 95% CI [0.01, 0.04]). This model of performance in the learning blocks is depicted in Fig. 2A. The BIC of the model that included repetition (10,234.7) was considerably lower than that of the one that did not (10,281.5), providing strong evidence of its improved fit.

The test block was also analyzed using a binomial GLME model (Fig. 2A). In the test block, categorization accuracy was above chance for prototypes in the delayed and early conditions (β_P_ = 2.492, *P* < 0.001, 95% CI [2.17, 2.84]; β_P_ = 2.010, *P* < 0.001, 95% CI [1.72, 2.32]) and for rule-following items in the delayed and early conditions (β_R_ = 0.996, *P* =  < 0.001, 95% CI [0.75, 1.25]; and β_R_ = 0.914, *P* =  < 0.001, 95% CI [0.67, 1.17], respectively). For exceptions, accuracy was above chance in the delayed condition (β0_E_ = 0.732, *P* < 0.001, 95% CI [0.49, 0.98]) but not in the early condition (β0_E_ = 0.132, *P* = 0.272, 95% CI [− 0.11, 0.37]). In the early condition, categorization accuracy was significantly higher for rule-followers than exceptions (β_R:E_ = 0.782, *P* < 0.001, 95% CI [0.54, 1.01]), and accuracy was higher for prototypes than rule-followers (β_P:R_ = 1.096, *P* < 0.001, 95% CI [0.72, 1.29]). The same patterns held in the delayed condition (β_R:E_ = 0.264, *P* = 0.035, 95% CI [0.02, 0.51] and β_P:R_ = 1.496, *P* < 0.001, 95% CI [1.17, 1.84], respectively). Categorization accuracy was significantly higher in the delayed condition than in the early condition for prototypes (β_P_ = 0.482, *P* = 0.034, 95% CI [0.04, 0.93]) and exceptions (β_E_ = 0.600, *P* = 0.001, 95% CI [0.26, 0.94]). There was no significant difference between conditions for rule-followers (β_R_ = 0.082, *P* = 0.645, 95% CI [− 0.267, 0.434]). Finally, there were also interactions between type and condition. The difference in categorization accuracy between exceptions and prototypes did not significantly change across conditions (β = − 0.015, *P* = 0.944, 95% CI [− 0.45, 0.42]), but the difference between exceptions and rule-followers was significantly smaller in the delayed compared to the early condition (β = − 0.510, *P* = 0.003, 95% CI [− 0.85, − 0.17]), and the difference between rule-followers and prototypes was significantly greater in the delayed condition (β = 0.495, *P* = 0.027, 95% CI [0.06, 0.94]).

The results from both the learning and test blocks indicate that manipulating trial order by delaying the introduction of exceptions significantly improved categorization accuracy of exceptions. As hypothesized, categorization accuracy for exception items improved more over time in the delayed condition than in the early condition. However, a post-hoc analysis of category A and B exceptions (EA and EB, respectively) revealed that this effect was not consistent for both exceptions, as shown in Fig. 2B. To contextualize the results that follow, we note that when we more closely compare EA and EB to members of their respective categories that share the same nondiagnostic dimension, an asymmetry emerges in the category structure. EA is closer to the prototype and rule-follower of its category that share the same nondiagnostic value than EB is to its nondiagnostic counterparts in Category B. It seems that, though this increased similarity to its own category members is driven by the non-diagnostic dimension, EA behaved more like a rule-follower; conversely, EB behaved more like an exception.

Learning block categorization accuracy did improve more over time for both EA (β = 0.028, *P* = 0.015, 95% CI [0.022, 0.063]) and EB (β = 0.042, *P* < 0.001, 95% CI [0.005, 0.050]) in the delayed condition compared to the early condition, indicating that our manipulation was indeed successful for both exceptions, but in the test block, the delayed condition was only associated with higher accuracy for EB (β = 1.573, *P* < 0.001, 95% CI [1.136, 2.019]); for EA, there was no significant difference between conditions (β = − 0.455, *P* = 0.047, 95% CI [− 0.909, − 0.004]). Moreover, accuracy at test was above chance for EA in both the early and the delayed conditions (β = 1.166, *P* < 0.001., 95% CI [0.80, 1.54] and β = 0.798, *P* < 0.001, 95% CI [0.43, 1.16], respectively); for EB, accuracy was below chance in the early condition (β = − 0.789, *P* < 0.001, 95% CI [− 1.14, − 0.43]) and above chance in the delayed condition (β = 0.862, *P* < 0.001, 95% CI [0.49, 1.23]). As these results indicate, category had a significant effect on accuracy in the early condition (β = 1.955, *P* < 0.001, 95% CI [1.60, 2.31]); accuracy was higher for EA than EB. This effect was not present in the delayed condition (β = − 0.063, *P* = 0.727, 95% CI [− 0.42, 0.29]). Condition had a significant effect on accuracy for EB (β = 1.651, *P* < 0.001, 95% CI [1.14, 2.16]); that is, accuracy was significantly higher in the delayed condition, but for EA, this effect was not significant (β = − 0.367, *P* = 0.166, 95% CI [− 0.89, 0.15]). Finally, there was a crossover interaction between category and condition: the difference in accuracy between EA and EB was significantly lower in the delayed condition (β = − 2.02, *P* < 0.001, 95% CI [− 2.52, − 1.51]). Participants in the early condition seemed unable to identify EB as an exception and instead attempted to categorize it according to the general category rules, leading to performance that was below chance; however, in the delayed condition, participants seemed better able to identify EB as an exception, leading to above-chance accuracy. These results seem to indicate that EA is behaving more as a rule-follower, and EB is behaving as an exception.

Participant reaction time (RT) was also recorded during the learning and test blocks to assess uncertainty throughout the learning process. Models analogous to the GLME models used to analyze categorization accuracy in the learning and test blocks were fit to reaction time data. The key findings from these analyses are as follows (see Supplement for complete results). In the learning blocks, RTs for prototypes were faster in the delayed compared to the early condition. When category-violating information was withheld, participants seemed better able to quickly categorize the items that defined category structure. Further, RT for exception items became faster with repetition in the delayed compared to the early condition. Consistent with the accuracy analyses, participants were able to rapidly identify and learn to categorize exceptions in the delayed condition relative to the early condition. Interestingly, RT at test was slower for rule-followers in the delayed compared to early conditions, which may reflect some uncertainty in overall category structure elicited by the delayed introduction of exceptions.

Results from the recognition memory task supported existing work on enhanced memory for exception items^2,5,29^. The complete analysis of this task is included in the Supplement. Notably, participants’ sensitivity to previously encountered stimuli was only above chance for exceptions in the delayed condition; moreover, sensitivity was significantly higher for exceptions than rule-following items in the delayed, but not early, condition. Further optimizing the recognition memory task used in this work by including lures that vary across both diagnostic and non-diagnostic dimensions may provide further insight into these effects.

Our behavioural results confirmed that delaying the presentation of exception items allows the learner to form a better understanding of the category structure, enabling them to subsequently identify and learn to correctly categorize exception items. To test whether these findings may be related to hippocampal computations, we next simulated our task using a neural network model of HC. We endeavoured to determine whether the model also predicted an advantage for exceptions and whether this advantage was specific only to EB. Such findings would provide novel computational evidence for the role of hippocampal learning systems in RPE tasks.

### Computational simulations

**Figure 3:**
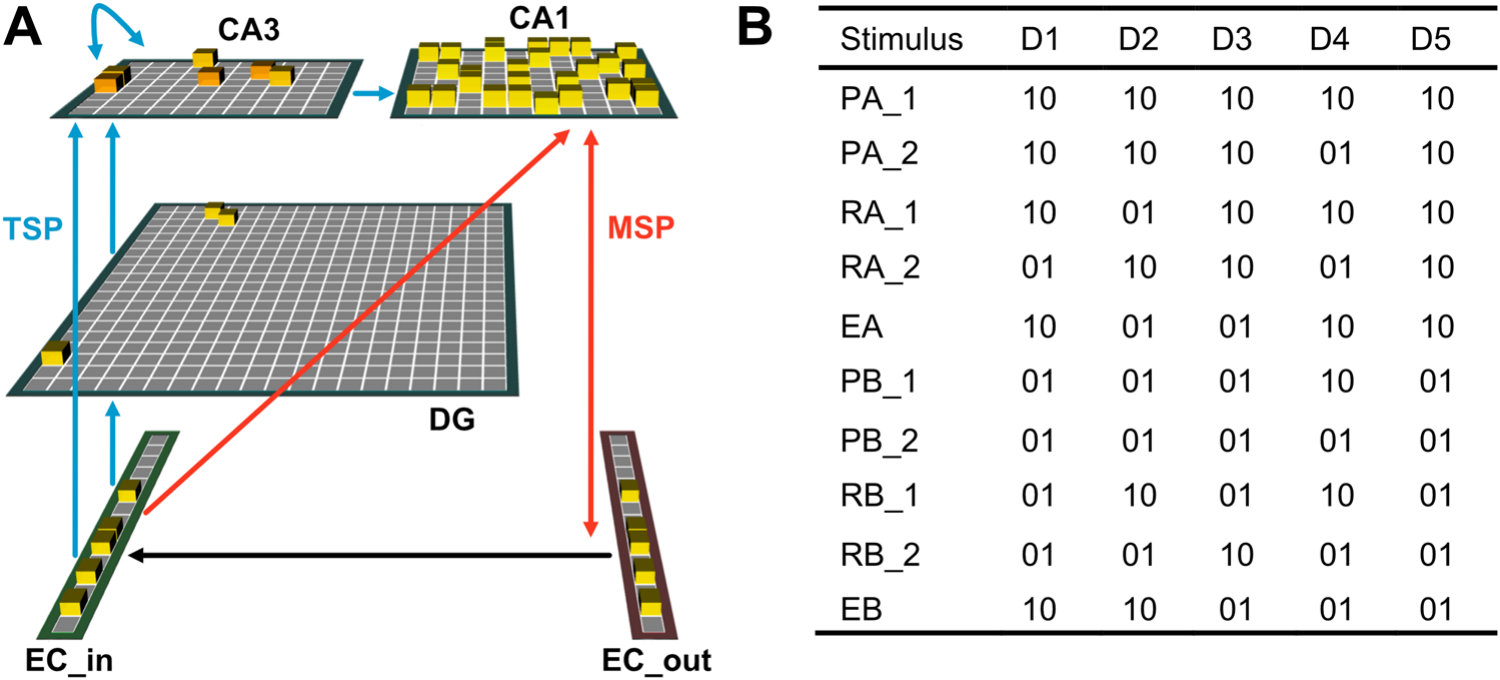
(**A**) A visualization of the hippocampal model used for computational simulations. The input layer, EC_in, represents superficial layers of entorhinal cortex. Information flows along two paths, the monosynaptic and trisynaptic pathways (MSP and TSP). Hidden layers represent hippocampal subfields dentate gyrus (DG), cornu ammonis 1 (CA1) and cornu ammonis 3 (CA3). The model acts as a simple encoder and attempts to replicate its input in EC_out, which represents deep layers of entorhinal cortex. EC_in also receives input from EC_out to simulate big-loop recurrence. (**B**) Vector notation for prototypes, rule-followers, and exceptions (P, R, and E, respectively) in categories A and B. Dimensions 1 through 4 represent the features outer petal colour, outer petal shape, inner petal shape, and central disc colour; dimension 5 indicates category. All features are binary and represented in a padded format in the model (i.e., ‘10’ or ‘01’).

To test how our sequence manipulation potentially targets HC computations and impacts neural representations, we simulated our task using an existing neural network model of HC and its subfields (Fig. 3)^15,25^. The model was trained using a learning sequence that matched either the delayed or early behavioural condition, then tested by presenting each of the 10 stimuli and recording output activation separately across the model layers. Notably, no parameter optimization or fits to behaviour were conducted in these simulations. Rather, the model, as formalized in prior work demonstrating its ability to account for statistical learning and associative inference behaviours^15^, was simply exposed to the same training sequences as the behavioural participants. The key question is whether such a model naturally accounts for the exception learning behaviour we observe, serving as a proof of concept implicating hippocampal encoding in complex category learning. To explore this question, 500 simulations, or batches, with random weight initializations were run for each sequence condition. Model accuracy for each stimulus was quantified by the relative difference in cosine similarity between the model’s output (*EC_out*) for the category dimension (*x*_*D5*_) and the target and nontarget categories using Luce’s choice axiom^30^ as shown in Eq. (1):

1 $$accuracy_{M} \left( x \right) = \frac{{cos\left( {x_{D5} , target_{D5} } \right)}}{{cos\left( {x_{D5} , target_{D5} } \right) + cos\left( {x_{D5} , nontarget_{D5} } \right)}}.$$

### Model learning performance

**Figure 4:**
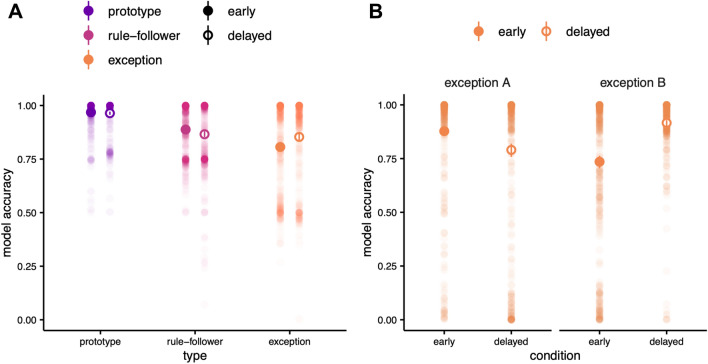
(**A**) Model categorization accuracy for all stimuli. Large points indicate the median of bootstrapped mean accuracies for stimulus types in the early (filled) and delayed (open) sequences. Error bars represent bootstrapped 95% confidence intervals. Small dots show average accuracy for each batch. The model results for exception items were consistent with behavioural findings: accuracy was significantly higher for exceptions in the delayed condition. (**B**) Exception results, separated by category. Again, the model findings were consistent with behavioural results: the advantage for exceptions in the delayed condition was driven by the category B exception.

The model’s categorization performance was analyzed using a GLME model with stimulus type and sequence condition as fixed effects and simulation batch number as a random effect (see “Methods” section). For exceptions, there was a significant effect of sequence condition. As with the behavioural findings, accuracy for exceptions was higher in the delayed condition than in the early condition (Fig. 4A; β_E_ = 0.047, *P* < 0.001, 95% CI [0.03, 0.07]). However, prototype accuracy was not significantly different in the delayed condition (β_P_ = − 0.004, *P* = 0.550, 95% CI [− 0.02, − 0.01]) and performance decreased for rule-followers in the delayed condition (β_R_ = − 0.022, *P* = 0.003, 95% CI [− 0.04, − 0.01]). Moreover, there were interactions between condition and type. The difference in categorization accuracy between exceptions and prototypes was significantly smaller in the delayed compared to early condition (β = − 0.051, *P* < 0.001, 95% CI [− 0.08, − 0.03]), as was the difference between exceptions and rule-followers (β = − 0.069, *P* < 0.001, 95% CI [− 0.09, − 0.04]), and the difference between rule-followers and prototypes did not significantly change across conditions (β = 0.017, *P* = 0.089, 95% CI [− 0.04, − 0.003]).

The model simulations capture the expected advantage for exception items in the delayed condition. The model categorized exceptions more accurately after exposure to the delayed sequence compared to the early sequence, demonstrating that the HC neural network is sensitive to learning sequence in a manner consistent with human learners. Recall that the behavioural results indicated an unexpected discrepancy between performance for EA and EB in the delayed condition. Importantly, this discrepancy was reflected in the model simulations (Fig. 4B). The advantage afforded to exceptions in the delayed condition was specific to EB (β_EB_ = 0.181, *P* < 0.001, 95% CI [0.15, 0.21]); performance was reduced in the delayed condition for EA (β_EA_ = − 0.087, *P* < 0.001, 95% CI [− 1.14, − 0.06]). Similar to the crossover interaction observed in participants’ test performance, model accuracy also demonstrated an interaction between condition and category (β = − 0.268, *P* < 0.001, 95% CI [0.23, 0.31]), whereby the advantage afforded to exceptions in the delayed condition was specific to EB.

The HC model accounted for both key behavioural findings related to exceptions; however, the model also predicted significantly higher performance for rule-followers in the early condition compared to the delayed condition. While participant data revealed no differences in the test performance for rule-followers, a post-hoc, qualitative inspection of trial-by-trial accuracy in the behavioural learning phase data was generally consistent with model predictions. Participant accuracy for rule-followers did drop when exceptions were introduced in the delayed condition. Specifically, rule-follower accuracy in the second learning block (i.e., when exceptions were first introduced) decreased from 0.68 in the first half to 0.62 in the second half, which may reflect a disruption in rule-follower performance when exceptions were introduced. Though participants seemed to resolve this discrepancy in accuracy by the end of learning, the RT analysis indicated increased RT for rule-followers in the delayed condition at test, reflecting persistent uncertainty. Current models of category learning such as SUSTAIN do not consider how highly salient, category-inconsistent information affects existing representations, and the insight into human behaviour provided by the hippocampal model should be a focus of future work.

### Model representational similarity analysis

With strong evidence of the HC model reflecting participant learning outcomes, we next interrogated the nature of the representations formed by the model during learning. Specifically, we conducted an exploratory representational similarity analysis on the activations of the hidden layers of the model corresponding to CA1, CA3, and DG hippocampal subfields. Previous work has indicated that medial temporal lobe representations capture both category structure and the similarity of exceptions to other stimuli^6^. We were interested in whether our delayed/early manipulation would impact how exceptions would be integrated into existing category knowledge reflected in the model’s hidden layers and whether exception representations would capture the discrepant results for EA and EB.

**Figure 5:**
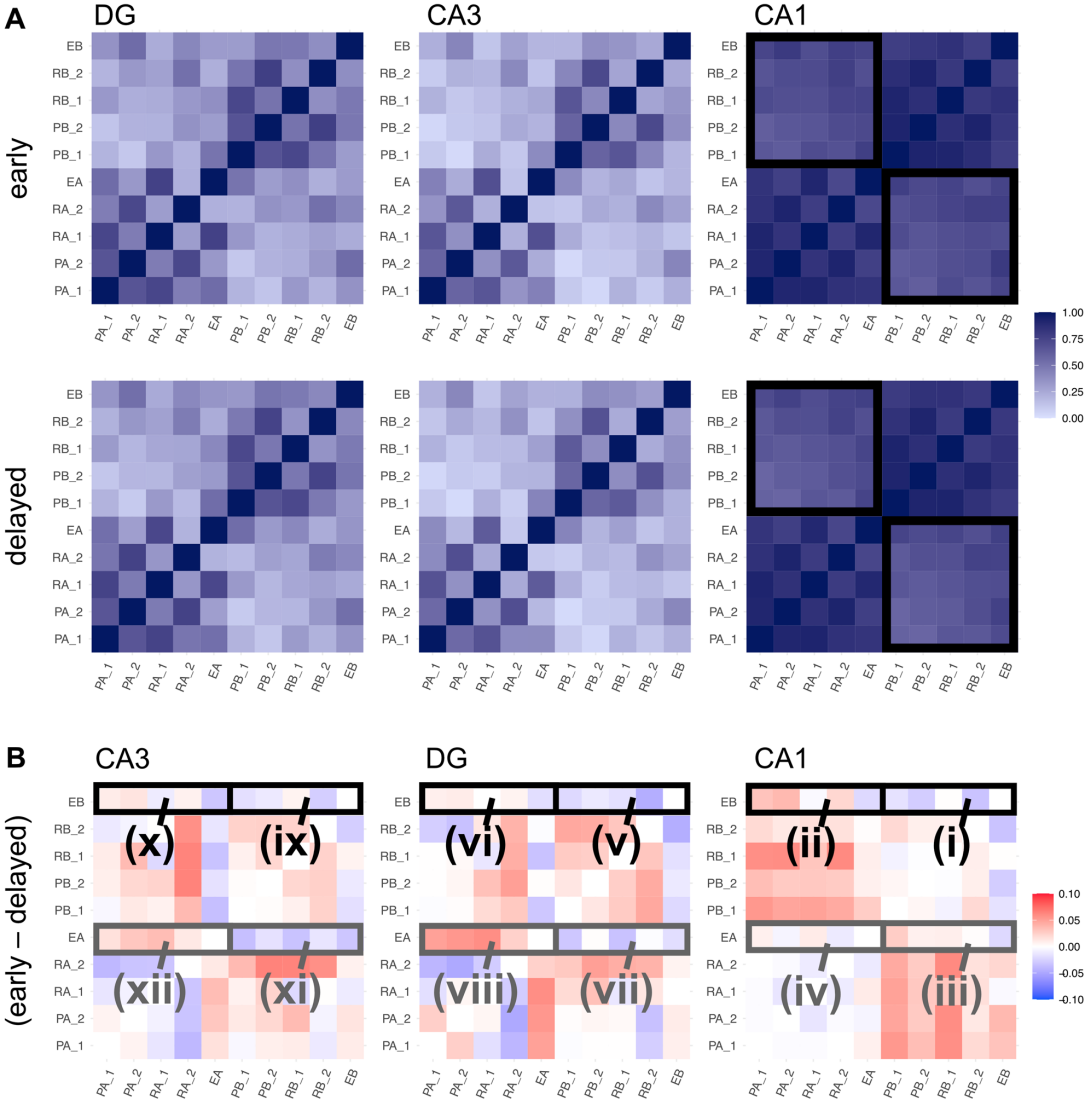
(**A**) Representational similarity analysis results, separated by condition and subfield. Rows and columns correspond to the 10 stimuli. Darker shades indicate higher similarity. In all subfields, higher intra-versus intercategory similarity is apparent. Category bounds are indicated in black in the CA1 grid. (**B**) The difference in representational similarity across conditions (early minus delayed). Red indicates higher similarity in the early condition; blue, higher similarity in the delayed condition. The red colour of the upper left and lower right quadrants of the rightmost plot indicates higher intercategory similarity in CA1 in the early compared to the delayed condition. Differences in exception representation are evident throughout the three subfields. EA is generally more similar to its own category members in the early condition and more similar to opposing category items in the delayed condition (these comparisons are outlined in grey), whereas EB is more similar to its own category members in the delayed condition and more similar to members of the opposite category in the early condition (outlined in black).

During the model’s testing phase, we recorded settled activation in the hidden layers of the network corresponding to CA1, CA3, and DG. In keeping with methods from related work^15^, we calculated the Pearson correlations between activations for each test item (i.e., between each of the 10 stimuli) in each of the layers. The results of this analysis are depicted in Fig. 5A, where darker shades represent higher representational similarity (the darkest squares, along the diagonal, correspond to a stimulus’ correlation of 1 with itself). The overlapping representations of CA1 are reflected in the overall shade of the grids corresponding to this subregion, which are darker than those of CA3 and DG, consistent with these regions’ sparser representations. The representational similarity of CA1 can also be visually clustered into zones: the darker lower left and upper right quadrants of the CA1 grids indicate higher intracategory similarity, whereas the lighter lower right and upper left quadrants indicate lower intercategory similarity. In other words, representations of members in the same category are more similar, whereas those of members in opposite categories are less similar. Although representations in CA3 and DG are overall more distinct (i.e., lighter) than those in CA1, higher intra- versus intercategory similarity is still evident—albeit to a lesser extent than in CA1—which may suggest sensitivity to category structure in these subfields. These patterns reflect existing research on the role of HC’s subfields; however, visually detecting representational differences between layers in the early and delayed conditions is quite difficult. To render these differences more apparent, Fig. 5B presents the difference between representational similarity in the early and delayed conditions, as determined by subtracting the delayed representational similarity matrix from the early matrix for each of the three subfields.

In Fig. 5B, the colour red indicates higher similarity between two stimuli in the early condition compared to the delayed condition, and blue, higher similarity between two stimuli in the delayed condition compared to the early condition. In the grid of Fig. 5B corresponding to CA1, the red colour of the upper left and lower right quadrants denotes higher levels of intercategory similarity for stimuli in the early condition; that is, representations of members in opposite categories are more similar in the early condition compared to the delayed condition. Median intercategory similarities in the early and delayed simulations were 0.718 and 0.619, respectively; a Wilcoxon rank sum test indicated that this difference was statistically significant (W = 148,469, *P* < 0.001). A higher degree of similarity between members in opposing categories indicates a blurring of category boundaries, which may reflect the model’s reduced ability to distinguish exceptions, thus lowering exception categorization performance in the early condition.

To explore potential mechanisms for the differing behaviour of EA and EB in the early and delayed conditions, we next explored condition-related differences between inter- and intracategory similarity of EA and EB in each subfield. To quantify these differences, we calculated the normalized difference between the intra- and intercategory similarity for each exception and compared these values across conditions (for convenience, this measure is referred to as the category representational difference [CRD]):

2 $$CRD = \frac{{\left( {1 + \rho_{intra} \left( {EX} \right)} \right) - \left( {1 + \rho_{inter} \left( {EX} \right)} \right)}}{{\left( {1 + \rho_{intra} \left( {EX} \right)} \right) + \left( {1 + \rho_{inter} \left( {EX} \right)} \right)}}$$

where r is the average inter- or intracategory correlation and EX indicates EA or EB. A higher CRD would indicate that an item’s representation was more similar to its own category members and less similar to opposing members, which in turn should correspond to improved category performance.

Notably, the effect on the representations of the two exceptions across conditions does not seem to be consistent. Significant differences in inter- versus intracategory similarity across conditions were found for EB in CA1 and for EA in CA3. In CA1, the median CRD was significantly higher for EB in the delayed condition (early: 0.015, delayed: 0.024, W = 11,640, *P* = 0.002), as indicated by the blue colour of zone i and the red colour of zone ii in Fig. 5B. This shift in representation corroborates enhanced categorization for EB in the delayed condition. The median CRD for EA did not differ between conditions (early: 0.0216, delayed: 0.0271, W = 119,025, *P* = 0.229); these values correspond to zones iii and iv of Fig. 5B. In DG, the EB median CRD value was not significantly different in the delayed (0.015) versus early (0.134) conditions (W = 122,861, *P* = 0.679), nor was the EA early median CRD (0.033) significantly different than that of the delayed CRD (0.021, W = 132,423, *P* = 0.092; zones v–viii in Fig. 5B). In CA3, the EB early median CRD (0.009) was not significantly different from the delayed median CRD (0.017; W = 120,796, *P* = 0.416); zones ix and x of Fig. 5B). However, the EA early median CRD (0.040) was significantly higher than the delayed median CRD (0.026; W = 134,623, *P* = 0.026), corresponding to the blue colour of zone xi and the red colour of zone xii in Fig. 5B. In other words, in CA3, EA had higher intra- and lower intercategory similarity in the early compared to the delayed condition. Subfield characteristics that may have led to these differences are further explored in the discussion.

**Figure 6:**
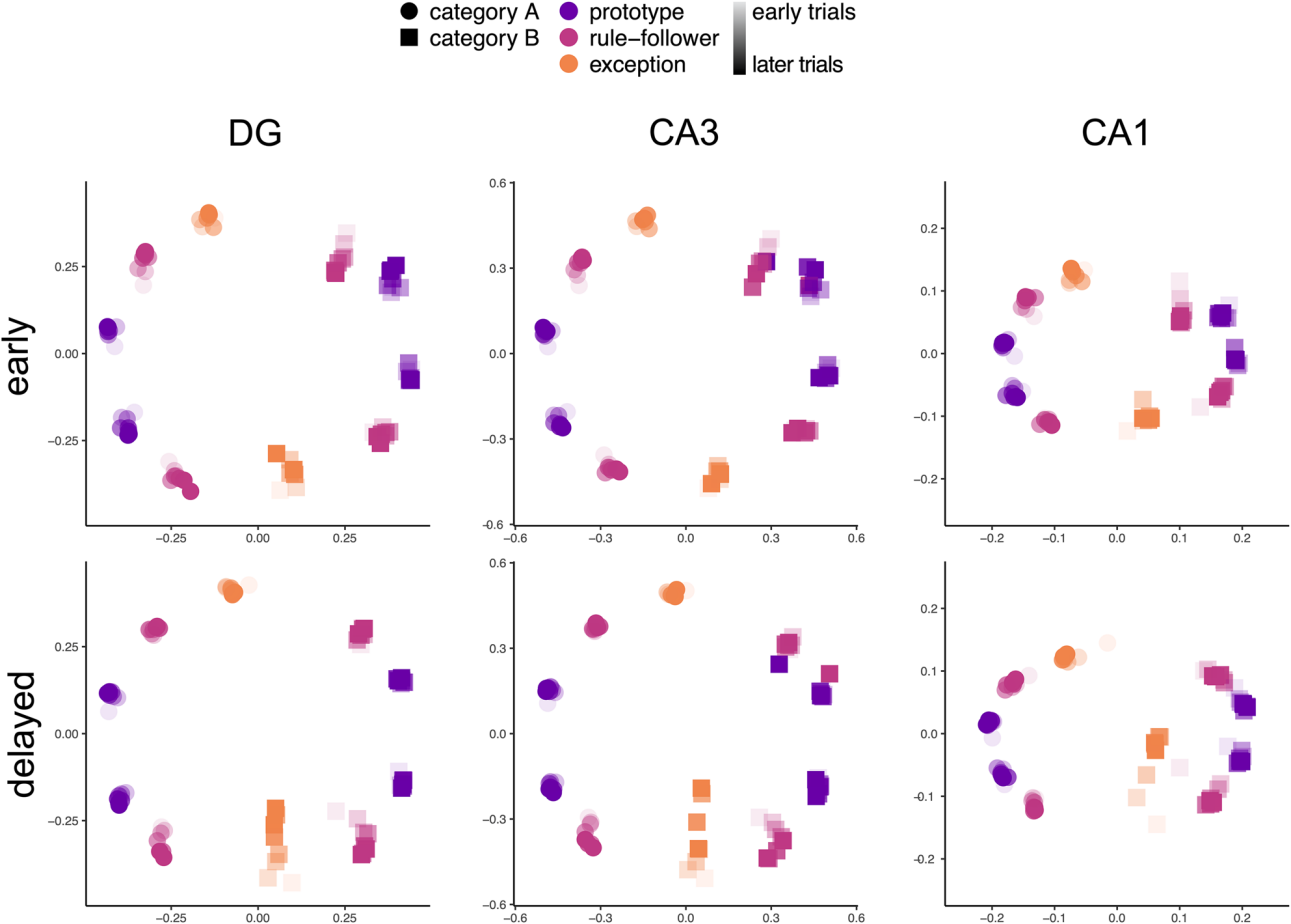
Sammon mapping projections of HC model subfield representations. Two-dimensional projections for stimulus representations within each subfield (columns) in the early (top) and delayed (bottom) sequences. Representations from tests at various time points throughout learning (early: trial 12, 36, 48, 72, 96, 120, 144; delayed: trial 24, 48, 60, 72, 84, 96, 120, 144) are depicted within the same plot with increasing opacity for later time points. The model clearly represents the space well after a limited number of trials. Category coding was apparent across subfields and sequences with category A stimuli (circles) on the left of the primary dimension and B stimuli (squares) on the right. Although stimulus locations were fairly consistent across subfields, a large shift was observed for exception B (orange square) across learning trials in the delayed condition. This shift was not observed for exception A.

To explore how inter- and intracategory differences for EA and EB emerged across the early and delayed conditions throughout learning, we used a Sammon mapping to project the representational similarity space derived for each subfield and condition into a two-dimensional space (Fig. 6). This approach has previously been used to explore information encoded in neural representations^6^. Sammon mappings were computed for representations at different timepoints throughout the model’s learning process (results were computed after early trials 12, 36, 48, 72, 96, 120, and 144 and delayed trials 24, 48, 60, 72, 84, 96, 120, and 144; the trial intervals varied between conditions to capture changes in representations when exceptions were first introduced). All Sammon mappings had a stress values of less than 0.035, indicating an excellent fit^31^. Procrustes transformations were used to align the Sammon mappings at different timepoints from each condition to the end-of-learning representations (i.e., after 144 trials) to facilitate comparison. In Fig. 6, increasing trial number is indicated by increasing opacity. EA and EB are generally located closer to X = 0 in this representational space, whereas prototypes and rule-followers are clustered by category at opposite ends of the space. The Procrustes transformation was successful in aligning most stimuli across conditions, indicating that encoding of category structure was consistent within subfields. However, in the delayed condition, EB representations in CA1 shift considerably towards EB’s own category members. Manipulating learning sequence clearly impacted changes in how exceptions were stored in relation to other items over time, but a large shift was not observed for EA across conditions.

## Discussion

Our aim was to explore how learning sequence impacts categorization performance in an RPE task. We provided behavioural evidence that delaying the introduction of exceptions significantly improved participants’ ability to categorize certain items. We hypothesized that this delay was advantageous because it targeted hippocampal learning systems: in MSP, early exposure to exclusively rule-following items would have enabled better encoding of general category structure; when exceptions were then introduced, increased mismatch signalling would precipitate the distinct encoding of exceptions in TSP. We used a model of HC to provide novel computational evidence of this proposed hippocampal learning mechanism. Notably, this model, which had previously been optimized for episodic and statistical learning, replicated behavioural results for exception categorization. The model also captured an unpredicted discrepancy in the effect of our manipulation on each of the two exceptions: enhanced categorization was observed for one exception, but not the other, in a manner consistent with subtle differences in the exceptions’ relative similarity to the other stimuli. Further analysis of the model’s hidden layers revealed unique changes in the representational similarity of the two exceptions that corroborated the model’s predictions.

Though the structure of the task used in this work is not universally applicable to all categories that could be defined as “rule-plus-exception,” our work presents key evidence of how learning systems within hippocampus encode new experiences in a manner dependent on previously encountered information. The advantage afforded to delayed exceptions may be explained by work on schema violation. Evidence of memory advantages for schema-violating information spans several literatures, and advantages seem to increase with the degree of schema violation^1–4^. It follows that enhancing this memory advantage may have been beneficial for correct categorization. Introducing exceptions after participants had a well-established understanding of category structure would enhance the surprise elicited by exceptions, thus facilitating the flexibility necessary to form conjunctive representations that would improve subsequent categorization. Existing work has indicated that learning sequence may impact performance in RPE tasks. Notably, work by Mathy and Feldman^20^ demonstrated that presenting rule-following items before exception items impacted overall categorization performance. However, they only delayed the introduction of exceptions from one category, referred to as the positive category (trials both rule-following and exception stimuli from the opposing category were interleaved with the positive trials), and they examined how overall learning was impacted, rather than exploring specific effects on exception items. Moreover, Davis et al.^8^ noted that presenting all rule-following items before exceptions sped up learning and reduced the number of non-learners in their RPE task, no quantification of these effects was provided. Our work is the first to manipulate learning sequence to directly target categorization of exception items.

Because forming distinct conjunctive representations is essential to the correct categorization of exceptions in an RPE structure, a function which has been previously attributed to HC^12,13^, we chose to further explore the behavioural findings using a neural network model of HC. Our aim was to evaluate, as a proof of concept, whether this model’s hippocampal encoding functions can demonstrate the type of exception learning effects we observed in human learners across different sequences, as has been done previously with other non-episodic memory behaviours^15^. Importantly, our behavioural learning results for exceptions were replicated in these computational simulations, without altering the model or conducting any parameter optimization. Our model findings support and extend existing evidence of HC’s role in category learning^5,8–11^. Studies on populations with limited HC function further emphasize this brain region’s importance to RPE learning. Individuals with underdeveloped or damaged HC exhibit impaired RPE learning, likely due to their reduced ability to form the requisite conjunctive representations^32^. Rodent work has also indicated that CA3 damage prevents the rapid formation of conjunctive representations, a capacity essential to exception learning^33,34^. Indeed, one recent study in young adults demonstrates a distinct association between structural measures of hippocampal pathways and RPE learning such that the number of streamlines connecting CA3 and CA1 (i.e., a key connection in the trisynaptic pathway) relates to an individual’s ability to learn exceptions^35^. Here we demonstrate learning in healthy young adults can be influenced by the way in which information is presented. We also provide novel evidence that a physiologically informed model of HC and its subfields has the capacity to learn an RPE category structure and that the way in which information is stored in this model is altered by learning sequence. Future work should explore how individual differences in hippocampal anatomy might affect performance in this task^35^ and constrain model predictions of learning behaviour.

The hippocampal neural network model captured patterns in exception categorization accuracy; however, its performance for rule-following and prototype stimuli did not align as closely with behavioural results. It should be noted that HC is just one brain region involved in the complex processes of category learning^7,36^, so one would not expect a neural network that only models hippocampal encoding to perfectly capture human behaviour. Indeed, our use of Luce’s Choice Axiom to transform model outputs into choice probabilities is an oversimplification of the decision-making process. Moreover, it has been demonstrated that several extant cognitive models of category learning^26,27,37^ account for RPE learning behaviour likely better than the current HPC model. However, the aim of the present work was not to identify the best fitting cognitive or neural model that best captures our behavioural results. Rather, our goal was, as a proof of concept, to explore whether a physiologically inspired model of hippocampus designed to account for key behavioural markers in episodic memory and statistical learning^15^ would naturally account for RPE category learning. By doing so, our work is poised to bolster our understanding of hippocampus to an encoder that not only stores episodic memories but also integrates new information with existing memories by extracting commonalities and differences to dynamically create new concepts^38^. The current findings also motivated future work that directly compares cognitive and neural models to characterize comprehensive theories of human category learning that bridge levels of analysis.

In addition to supporting behavioural findings related to exceptions, the model simulations shed light on how this manipulation may have targeted hippocampal representations at a subfield level. Representational similarity analyses on model representations exposed subtle but important differences in representations across conditions. In CA1, there were higher levels of intercategory representational similarity in the early compared to the delayed condition. Carvalho and Goldstone’s sequential attention theory^39^ posits that a blocked learning sequence, where the learner is presented with blocks of items from one category, causes them to attend to intracategory similarities, whereas an interleaved design that alternates between items from two categories draws their attention to intercategory differences. However, our model results indicate that inter- and intracategory differences can also be influenced by when schema-violating stimuli are introduced. Moreover, the computational model used for these simulations has no explicit attentional mechanism. Attentional tuning to stimulus dimensions is a key mechanism in many successful category learning models^26,37,40^, often providing the computational flexibility necessary for learning and representing complex category structures^41^. That the HC model leveraged here has no explicit mechanism of attention yet successfully learns in an RPE task as well as other similarly complex category-like structures^11,15^ suggests such dimension-weighting attention may be unnecessary or replaced by HC encoding functions in certain learning contexts. A theoretical reconciliation of these models awaits future study.

A notable finding from both our behavioural results and computational simulations was the discrepancy between exceptions. Further consideration revealed that this discrepancy was likely a consequence of the nondiagnostic fourth dimension of our category structure, inner circle colour. Due to differences in exception similarity to category members sharing the same non-diagnostic dimension, EB could be considered more “exceptional” than EA, which would have afforded it a greater advantage in a learning sequence predicted to emphasize exceptions. Though behavioural results showed an advantage for both exceptions during learning, it seems that in human and model, the exceptions’ overall similarity to previously encountered exemplars impacted memory and categorization, even when that similarity was due to a nondiagnostic dimension. Although pinpointing the specific factors leading to the discrepancy between exceptions in the current paradigm is an open question, that model-based predictions of this divergence matched those of human learners provided serendipitous evidence in support of HC computations underlying RPE learning. Indeed, analysis of inter- versus intracategory similarity in the early compared to the delayed condition revealed that in CA1, EB representations were more like its own category members and less like opposing category members, reflecting better integration of EB with its own category members when its introduction was delayed. Looking forward, these findings motivate a role for CA1 in exception learning; future work characterizing neural function driving exception learning should target mismatch signalling and neural representations in CA1.

A final contribution of this work is the methods we have employed to better understand what is happening in the hidden layers of our model. A common criticism of neural network models is their “black box” nature^42^—it is difficult to understand the factors that contribute to a neural network classifier’s decisions. Exploring the representations of various stimuli in different hidden layers of a model may help to disentangle this decision-making process and provide key predictions for investigating neural representations in the learning brain.

Overall, this work demonstrates that performance on an RPE category learning task can be modulated by manipulating learning sequence. We also provide novel computational evidence of HC’s sensitivity to this manipulation and use representational similarity analysis to explore the impact of trial sequence on inter- and intracategory representational similarity. The experiments presented here serve as a starting point for future studies to further explore how HC and its pathways are implicated in category learning tasks and in cognition more broadly^7,38,43^.

## Methods

### Behavioural study

#### Participants

All participants were University of Toronto students who received course credit for participating. Data were collected in lab and, as necessitated by COVID-19-imposed restrictions, online. There were 49 in-lab participants (37 females; mean age 19.1 years, SD 3.3 years) and 44 online participants (20 females, 2 other; mean age 19.9, SD 1.0 years). In total, 93 participants completed the experiment. All procedures were approved by and conducted in accordance with the University of Toronto’s Research Ethics Board and all participants provided informed consent.

Participants were excluded from the analysis if they failed to achieve an accuracy of over 0.75 for any stimulus type in at least one of the learning or test blocks or if over 20% of their reaction times fell outside the range [0.15 s, 2 s]. Based on these criteria, 10 online participants and two in-lab participants were excluded, resulting in a total of 81 participants included in the analyses. Data from included participants were further preprocessed to exclude any responses less than 0.15 s or greater than 2 s (7.1% of all trials were excluded).

#### Stimuli

Throughout the experiment, participants viewed 10 images of flowers. Flower stimuli had four binary features: outer petal colour, outer petal shape, inner petal shape, and central disc colour (Fig. 1a). The central disc was determined to be least salient in a norming study and as such was chosen to be nondiagnostic and varied randomly between stimuli. Category structure was assigned using Shepard et al.’s (1961) Type 3 problem^24,44^, as shown in Fig. 1b. Stimuli were classified as prototypes (maximally dissimilar across categories), rule-followers (more similar to their category prototype than to the other category prototype), and exceptions (more similar to the prototype of the opposite category). The experiment included four prototypes (two in each category for each value of the nondiagnostic feature), four rule-followers, and two exceptions (for which the nondiagnostic feature varied randomly), resulting in a total of 10 stimuli.

#### Procedure

Participants completed three learning blocks, each with 48 trials. Full feedback was provided after each trial (Fig. 1b). Participants were randomly assigned into one of two conditions. In the “early” condition, participants were first introduced to exceptions in block one, and in the “delayed” condition, participants were not exposed to exceptions until the second learning block (Fig. 1c). Participants saw two times more prototypes than rule-followers and exceptions to anchor each category. Further, the first eight trials of the first learning block consisted of only prototypes in both conditions to expose participants to all possible feature values of the stimulus space. Following the learning blocks, participants completed a test block with 48 trials. In this block, participants saw an equal number of prototypes, rule-followers, and exceptions and did not receive feedback after each response. Participants also completed a recognition memory task in which they were shown previously encountered and novel stimuli and had to identify these stimuli as “old” or “new.” This task was intended to test for enhanced recognition memory for exceptions, as found in existing work^5^. The complete results from this preliminary investigation have been included in the Supplement. In-lab participants were also asked to describe their categorization strategies, but these strategies were inconclusive (for example, participants noted focusing on aspects of the stimuli unrelated to the categorization task, such as the number of green leaves on the flowers, but still met learning criteria), and there were no apparent differences between self-reported strategies across conditions. These responses have been uploaded to the OSF project associated with this work (https://osf.io/gner5).

#### Analysis

To account for the higher number of prototypes versus other stimulus types in the three learning blocks, only the first 36 repetitions for each type were included in the analysis. A binomial generalized linear mixed-effects (GLME) model was fit to the learning data (lme4 ver. 1.1–26, R ver. 4.0.4) to predict trial-by-trial accuracy. Because the response data were binary and thus not normally distributed, accuracy was modelled using a binomial regression with a logit link function. Inputs to the model were trial scores (0 for incorrect, 1 for correct). A GLME model was fit to the data that included stimulus type and condition as fixed effects and participant as a random effect to assess changes in average accuracy across all three learning blocks. To explore how learning was impacted by experience, a second model was then fit to the data that also included repetition as a fixed effect. Repetition was defined as the number of times a participant had seen a given stimulus type. The test block was analyzed using a GLME model that was identical to the base model of the learning block. Performance in the learning and test blocks was visualized by plotting average accuracy for each stimulus type in each of the three learning blocks and the test block (Fig. 2A). Performance for each exception was also visualized separately by plotting average accuracy for exception trials in each of three blocks of 12 trials (“repetition block” in Fig. 2B). Similar analyses were also conducted on reaction time; these results have been included in the Supplement.

### Model simulations

#### Overview of model architecture

To further study the impact of sequence on category learning, the RPE task described above was simulated using a neural network model of HC. This model was adapted from recent work^15^ and was run using Emergent7^45^, ver. 8.5.2. A simplified explanation of the model’s architecture is as follows: input patterns in the form of numerical arrays are presented to the model via its input layer, EC_in (which represents superficial layers of EC). During training, the model acts as an encoder and learns to replicate the pattern presented to EC_in in its output layer, EC_out (which represents deep layers of EC). The model accomplishes this goal by adjusting the weights of connections between its hidden layers through a combination of error-driven and Hebbian learning in cycles that reflect hippocampal theta oscillation^46^. The model’s hidden layers represent subfields DG, CA3, and CA1. Each layer of the model contains a grid of several units with activity levels ranging from zero to one. These units represent populations of neurons. Moreover, each layer has physiology-based properties. For example, model layers CA3 and DG have high within-layer inhibition, leading to the sparse representations characteristic of their corresponding neural subfields. Connections between layers mimic the flow of information along TSP and MSP, and the learning rate of TSP is also faster than that of MSP. The model is shown in Fig. 4. The model has free parameters that allow the user to adjust the strength of connections between CA3 and CA1 and EC_in and CA1 to simulate white matter lesions. Because this study involved healthy young adults, the fully connected values from Schapiro et al. were used^15^. A depiction of the model and its subfields and connections is included in Fig. 3A.

#### Training and testing

The flower stimuli were first transformed into vectorized input patterns according to a padded coding scheme. Each input vector had five pairs of units, and each pair represented a feature dimension. The first four pairs (units 0 to 7) corresponded to the four binary-valued dimensions, and the final pair (units 8 and 9) indicates category label. Each unit in a pair represents one of two possible values for a given dimension, so only one unit in a pair will be active (i.e., have a non-zero value) for a stimulus. For example, pointed petals may be coded as “01”, round petals, by “10”. In vector notation, the prototype for category A is therefore represented as “1010101010.” Vector notations for each stimulus are included in Fig. 3B.

Two training sequences were created for the model that corresponded to the early and delayed conditions of the behavioural experiment. The number of stimuli and trial order presented to the model in each condition were identical to the sequences of the behavioural experiment, but the learning task was not separated into blocks. In a training epoch (144 trials), stimuli were presented to EC_in sequentially. With each trial, the model updated its connection weights to replicate the input pattern in its output layer. After training, the model was tested: each of the 10 stimuli were presented to the model and its settled response (measured after 80 cycles) was recorded. No network weights were updated during test. The delayed and early sequences were each simulated 500 times (i.e., 500 batches were run) on randomly initialized networks. Batch number was included as a random effect in all analyses. At test, model accuracy for each stimulus was quantified using Luce’s choice axiom^30^ as shown in Eq. (1).

#### Analysis

A GLME model was used to assess model performance. Batch number was included as a random effect and type and condition as fixed effects. We also explored activation of the model’s hidden layers using representational similarity analysis. As in^15^, for each of the model’s hidden layers (CA1, CA3, and DG), we calculated the Pearson correlation between the activation of each of the 10 stimuli (Fig. 5A). To highlight the difference between conditions, we also calculated the difference between Pearson correlations for each condition by subtracting the delayed representational similarity matrix of each subfield from the corresponding early matrix (Fig. 5B). The subfield-level difference in inter- and intracategory similarity for EA and EB was quantified using Eq. (2). For each subfield, outputs of Eq. (2) were compared across the early and delayed conditions using a Wilcoxon rank sum test.

Finally, multidimensional scaling was used to visually depict the representational similarity between stimuli in a two-dimensional space^6^. We captured model activation patterns across subfields at different timepoints throughout the learning process to assess in more detail how representations changed when exceptions were first introduced in the early and delayed conditions. For the early condition, tests were run at 12-trial intervals for the first 48 trials and at 24-block intervals thereafter; in the delayed condition, tests were run at 12-trial intervals for trials 48–96 and at 24-block intervals otherwise. Representational similarity matrices based on Pearson correlation were constructed at each of these timepoints, and Sammon mapping^47^ was used to project the similarity relationships into a two-dimensional space. Finally, for each condition and subfield, each timepoint was aligned to the corresponding end-of-learning Sammon mapping using a Procrustes transformation^48^ to highlight similarities and dissimilarities in category structure throughout learning (Fig. 6).

## Supplementary Information

Supplementary Information.

## Data Availability

All behavioural data and end-of-learning model data are available at https://osf.io/gner5; details on the Emergent model used in these simulations can be found at^45^. Trial-by-trial model testing data are available from the corresponding author upon reasonable request.
