# Supplementary material for: Learning exceptions to the rule in human and model via hippocampal encoding
Source: Sci Rep. 2021 Nov 2;11:21429. doi: 10.1038/s41598-021-00864-9 (PMC8563716; doi:10.1038/s41598-021-00864-9)
Supplement: Supplementary file 1 — Supplementary Information. [file 41598_2021_864_MOESM1_ESM.docx]

**Learning exceptions to the rule in human and model via hippocampal encoding— Supplementary Analyses**

Emily M. Heffernan^*^, Margaret L. Schlichting, Michael L. Mack

Department of Psychology, University of Toronto, Toronto, ON, Canada
^*^emily.heffernan@mail.utoronto.ca

**Reaction Time**

Reaction time (RT) was recorded during the category learning and test blocks. RT can be used as a measure of uncertainty; specifically, slower reaction times correspond to increased uncertainty. Because RTs are not normally distributed, an inverse Gaussian general linear mixed-effects (GLME) model with an identity link function was used to assess how reaction time was impacted by repetition, type, and condition. In this and all RT analyses, participant was included as a random effect.

First, to explore the main effect of condition on reaction time for different stimulus types, a GLME model with condition and type as fixed effects was fit to the learning data. RTs were significantly faster for prototypes in the delayed compared to early condition (β = -0.0761, P = .002, 95% CI [-0.12, -0.03]); condition had no significant effect on RT for exceptions (β = -0.005, P = 0.836, 95% CI [-0.05, 0.04]) nor rule-followers (β = -0.038, P = .112, 95% CI [-0.09, 0.010]). In the early condition, reaction time for exceptions was significantly faster than that of rule-followers (β = -0.0318, P = .009, 95% CI [-0.06, -0.008]), and reaction time for rule-followers was significantly faster than that of prototypes (β = -0.039, P = .003, 95% CI [-0.07, -0.01]). These differences were not significant in the late condition (β = 0.001, P = .911, 95% CI [-0.02, 0.03] and β = -0.001, P = .916, 95% CI [-0.03, 0.02], respectively).

Repetition was then included as fixed effect to explore how reaction time changed over the course of learning. In the model that included repetition, reaction time for exceptions and prototypes became significantly faster with repetition in the early condition (β = -0.212, P < .001, 95% CI [-0.38, -0.02] and β = -0.252, P < .001, 95% CI [-0.48, -0.05], respectively). However, repetition had no significant effect on reaction time for rule-followers (β = -0.057, P = .357, 95% CI [-0.24, 0.12]). In the delayed condition, reaction time decreased with repetition for prototypes (β = -0.307, P < .001, 95% CI [-0.49, -0.14]), did not change significantly for exceptions (β = 0.092, P = .129, 95% CI [-0.09, 0.27]), and increased for rule-followers (β = 0.138, P = .019, 95% CI [0.02, 0.25]). Moreover, there was a significant interaction between repetition and condition for exceptions: reaction time increased with repetition in the delayed compared to early condition for exceptions (β = 0.304, P < .001, 95% CI [0.05, 0.54]); the interaction between condition and repetition was not significant for prototypes nor rule-followers (β = -0.054, P = .557, 95% CI [-0.31, 0.23]) and β = 0.008, P = 0.821, 95% CI [-0.05, 0.45], respectively). The BIC of the model that included repetition (5,350.4) was considerably lower than the model without repetition (5,361.8), providing very strong evidence of improved fit.

An inverse Gaussian GLME model with type and condition as fixed effects was fit to the test data. Condition only had a significant effect on reaction time for ruler-followers; specifically, reaction time was slower for these stimuli in the late condition (β = 0.0745, P = .016, 95% CI [0.01, 0.14]); reaction time did not significantly change between conditions for exceptions, nor prototypes (β = 0.029, P = 0.348, 95% CI [-0.03, 0.09] and β = 0.060, P = 0.055, 95% CI [-0.001, 0.12], respectively). In the early condition, type only had a significant effect when comparing exceptions to rule-followers; reaction time for exceptions was significantly slower (β = 0.048, P = .001, 95% CI [0.02, 0.08]). Reaction time for prototypes did not differ from rule-followers (β = 0.021, P = .128, 95% CI [-0.005, 0.05]). In the delayed condition, reaction time was not significantly affected by type – that is, reaction times for exceptions did not differ significantly from rule-followers (β = 0.003, P = .863, 95% CI [-0.03, 0.03]), nor rule-followers from prototypes (β = 0.006, P = .703, 95% CI [-0.02, 0.04]).

The faster reaction time for prototypes in the learning blocks may reflect the relative ease with which participants were able to encode the general category structure when exceptions were withheld. The increased reaction time for rule-followers at test and the interaction between repetition and condition for exception reaction time during the learning blocks may be indicative of increased uncertainty in the delayed condition.

**Recognition Memory**

Following the test block, participants completed a recognition memory task to explore whether participants exhibited enhanced recognition of exception items. On each trial, participants were shown a flower stimulus for two seconds and had to identify whether that stimulus was old or new. Trials contained either exceptions and rule-followers from the learning and test blocks or lures, which differed from their exception and rule-following counterparts along the non-diagnostic dimension.

Signal detection theory was used assess recognition memory for exception items. For each participant, d prime and criterion values were calculated for exception and rule-following stimuli. A general linear model with type and condition as predictors was fit to the d prime data. An ANOVA indicated that only the effect of type was significant, (*F*(1,160) = 5.192, MSE = 2.004, P = .024). There was no effect of condition (*F*(1,160) = 0.195, MSE = 0.075, P = .660) nor an interaction (*F*(1,160) = 0.282, MSE = 0.109 , P = .596). The effect of type seems to be driven by above-chance sensitivity to old exceptions in the delayed condition (β = 0.254, P = 0.010, 95% CI [0.06, 0.44]); no other comparisons were significant.

An identical GLM model was fit to criterion values. The mean criterion values for exceptions and rule-followers were significantly less than zero in the early and delayed conditions (for exceptions, β = -0.592, P < .001, 95% CI [-0.77, -0.41] and β = -0.578, P < .001., 95% CI [-0.75, -0.40]; for rule-followers, β = -0.473, P < .001, 95% CI [-0.65, -0.30] and β = -0.426, P < .001, 95% CI [-0.60, -0.25] in the early and delayed conditions, respectively), indicating a liberal response bias (that is, participants were more likely to respond “old”). This bias was likely due to the high similarity between stimuli. Condition had no effect on criterion for exceptions nor rule-followers (β = 0.014, P = .911, 95% CI [-0.24, 0.26] and β = 0.047, P = .714, 95% CI [-0.20, 0.30], respectively). There were no significant interactions between type and condition (β = -0.03, P = .856, 95% CI [-0.39, 0.32]).

The finding of greater memory sensitivity for exceptions in the delayed condition matches predictions from existing work on enhanced memory for surprising information^1^ and is consistent with our predictions; however, in total, our findings are mixed with only a single, limited significant effect for better exception memory in the delayed condition. The current recognition memory task included only six lure stimuli that varied across only the non-diagnostic dimension which limited our ability to characterize memory behaviour; future versions of this task should include stimuli that vary across both diagnostic and non-diagnostic dimensions.
